# Advanced magnetic resonance imaging techniques for structural and functional assessment of salivary glands

**DOI:** 10.2478/raon-2026-0025

**Published:** 2026-05-14

**Authors:** Jernej Vidmar, Andrej Vovk

**Affiliations:** 1 Institute of Physiology, Faculty of Medicine, University of Ljubljana, Ljubljana, Slovenia; 2 Centre for Clinical Physiology, Faculty of Medicine, University of Ljubljana, Ljubljana, Slovenia

**Keywords:** salivary glands, xerostomia, MRI, perfusion imaging, diffusion imaging, hyperbaric oxygen

## Abstract

**Background:**

Diagnostic paradigms for salivary gland disorders have undergone a radical transformation, shifting from purely morphological descriptions toward multi-parametric tissue characterization. This review evaluates the clinical efficacy of advanced magnetic resonance imaging (MRI) techniques, specifically quantitative T2 mapping and arterial spin labelling (ASL), in order to provide objective biomarkers for glandular pathophysiology. The use of pseudocontinuous ASL (pCASL) for non-invasive perfusion assessment and multi-dynamic multi-echo (MDME) sequences for quantitative mapping was highlighted in a methodical synthesis of recent research. Quantitative T2 mapping demonstrates high diagnostic accuracy in identifying glandular dysfunction; patients with hyposalivation exhibit significantly elevated T2 relaxation times (mean 91.85 ± 8.24 ms) compared to healthy controls (mean 80.69 ± 6.42 ms, p < 0.0001). ASL imaging reveals that parotid glands in Sjögren’s syndrome are hyperemic at rest (59.2 ± 22.8 mL/min/100g) but shows dysfunctional microvascular regulation after stimulation. Furthermore, apparent diffusion coefficient (ADC) values effectively differentiate pleomorphic adenomas (> 1.4 × 10–3 mm^2^/s) from malignant lesions.

**Conclusions:**

Advanced MRI provides robust, non-invasive metrics for monitoring radiation-induced damage and assessing the restorative impact of hyperbaric oxygen therapy (HBOT). By facilitating the shift to precision radiology in head and neck medicine, these technologies greatly improve patient quality of life and therapeutic planning.

## Introduction

The clinical management of head and neck pathologies requires increasing precision, especially in the early identification and characterization of salivary gland diseases. These glands, essential for oral homeostasis, digestion, and the protection of the oral mucosa, are frequent targets of inflammatory, autoimmune, and neoplastic processes. Over the past decade, the diagnostic paradigm has shifted from basic morphological description to a complex multiparametric characterization that includes structural, cellular, and functional analysis.^1^ This transition was driven by the limitations of traditional imaging, which often struggles to distinguish early stages of parenchymal degeneration from non-pathological changes associated with aging or systemic influences.^1^

The major salivary glands (parotid, submandibular, and sublingual) are organs with a complex histological structure. They consist of acinar cells responsible for secretion, ductal cells that modify primary saliva, and myoepithelial cells that aid in its expulsion. The diversity of these cell types and their varying susceptibility to external factors, such as ionizing radiation, viral infections, or autoimmune attacks, necessitate imaging methods that extend beyond the visualization of gross anatomical irregularities.^2^ Conventional techniques such as high-resolution ultrasound (HRUS) and computed tomography (CT) remain essential for initial lesion delineation and the detection of sialoliths.^2^ However, they are inherently limited in providing specific functional information about the state of the acinar parenchyma or microcirculation.^3^

Magnetic resonance imaging (MRI) is considered the gold standard in head and neck radiology due to its superior soft-tissue resolution and its unique ability to demonstrate perineural invasion (PNI), a critical prognostic factor in malignant salivary tumors.^4^ Nevertheless, conventional T_1_- and T_2_-weighted sequences rely on qualitative analysis of signal intensity, which is subjective and highly dependent on scanner hardware and sequence settings. This limitation has led to the development of quantitative methods that generate absolute physical values instead of relative grayscale intensities.^5^ Among these, quantitative T_2_ mapping and arterial spin labelling (ASL) stand out for their ability to noninvasively measure tissue hydration and microvascular perfusion.^6^

The review aims to provide a comprehensive evaluation of advanced imaging techniques for the structural and functional assessment of salivary glands. Special emphasis is given to the clinical utility of quantitative mapping in patients with hyposalivation, perfusion characterization, and the use of these methods in monitoring radiation induced injuries and therapeutic response to hyperbaric oxygen therapy (HBOT).^7^ By integrating these advanced radiological parameters into clinical practice, diagnostic accuracy can be improved, enabling more effective therapeutic planning and personalized management.

## Anatomical and pathophysiological framework

Salivary glands are highly specialized exocrine organs, and their structural integrity is a primary determinant of oral health. The parotid gland, the largest of the major glands, primarily contains serous acinar cells, while the submandibular and sublingual glands are mixed, containing both serous and mucous cells.^2^ The ductal system is also complex, involving intercalated, striated, and excretory ducts that modify the electrolyte composition of saliva.^2^ Understanding this architecture is crucial for interpreting advanced imaging, as different pathologies target specific histological components.

Radiation-induced damage, for example, primarily affects the radiosensitive acinar cells.^8^ Even at relatively low doses, ionizing radiation causes plasma membrane disruption and triggers apoptosis, leading to a rapid decline in saliva production.^8^ This process is progressive; acute inflammation and edema are followed by chronic fibrosis and fatty replacement of the parenchyma, a transition that can be objectively tracked using T2 mapping and fat-fraction analysis.^3^ Similarly, Sjögren’s syndrome involves an autoimmune attack on the glandular epithelium, characterized by focal lymphocytic aggregates that disturb normal microvascular regulation, as detectable by ASL perfusion imaging.^6^

In neoplasms, the histological diversity of salivary tissues gives rise to a wide variety of tumors with overlapping radiological features.^9^ Pleomorphic adenomas (PAs) are characterized by an abundant myxoid stroma, which provides high water mobility and characteristic mapping signatures.^10^ Warthin’s tumors (WTs), by contrast, feature a dense lymphoid component that restricts water diffusion, despite their benign nature. Distinguishing these from malignant lesions requires a multiparametric approach that combines assessments of cellularity, vascularity, and tissue stiffness.^11,12^

## Technical foundation of quantitative MRI

Traditional MRI provides images that are essentially “signal intensity weighted,” with contrast determined by the interplay of T_1_, T_2_, and proton density. While sufficient for anatomy, these images do not permit direct measurement of tissue properties.^9^ Quantitative MRI (qMRI) represents a paradigm shift toward using the scanner as a measuring device to obtain absolute physical constants.^9^

In *T*_2_ *mapping*, T_2_ relaxation, or transverse relaxation, refers to the process in which transverse magnetization components lose phase coherence. This process is primarily driven by dipole-dipole interactions between spins and local magnetic field inhomogeneities.^9^ In biological tissues, the T_2_ value is extremely sensitive to the concentration of free water and molecular mobility.^3^ Inflammation and oedema increase the amount of freely moving water molecules, which leads to longer T_2_ relaxation times. In contrast, the presence of macromolecules in fibrosis or the restricted environment of fatty tissue can lead to distinct shifts in T_2_ values.^13^

The standard method for T_2_ mapping involves acquiring multiple images at different echo times (TE) and fitting the signal decay to an exponential model.^14^ The mono-exponential decay function is typically used:

$$\text{S}(\text{TE})=\text{S}(0)\cdot {\text{e}}^{-\text{TE}/\text{T}2}$$

Where S(TE) is the signal intensity at a given TE, S(0) is the equilibrium signal (related to proton density), and T_2_ is the relaxation time.^14^ In clinical practice, acquiring multiple echoes traditionally required long scan times.^3^

*The multi-dynamic multi-echo (MDME) sequence* (mDIXON-Quant on Philips) has revolutionized quantitative MRI by enabling simultaneous quantification of T_1_, T_2_, and PD in a single acquisition.^3^ This sequence uses a combination of saturation pulses and multiple echoes to efficiently sample the relaxation curves efficiently. The resulting data can be processed with “synthetic MRI” software to generate any weighting (e.g., T_1_-weighted, T_2_-weighted, STIR) retrospectively, while providing parametric maps of the underlying physical values.^3^ This technology significantly reduces scan time, often by half, making it practical for the head and neck region, where motion and patient discomfort are common concerns.

*Arterial spin labelling* (ASL) is a powerful technique for non-invasively measuring tissue perfusion (salivary blood flow, SBF).^6^ It avoids the risks associated with gadolinium-based contrast agents, such as nephrogenic systemic fibrosis and allergic reactions, and allows for repeated assessments in short succession.^14^

Pseudo-continuous ASL (pCASL) is the recommended method for head and neck perfusion imaging. It utilizes a train of short radiofrequency (RF) pulses combined with a magnetic field gradient to induce flow-driven adiabatic inversion of blood spins as they pass through a labelling plane.^15^ This “labeled” blood serves as an endogenous tracer. After a post-labelling delay (PLD), carefully selected to allow the blood to reach the capillary exchange site, the target image is acquired. The difference between the control and labeled images provides the perfusion signal.^15^

Quantification of salivary blood flow (SBF) requires normalization by the Signal Intensity of a proton density-weighted reference image (SIPD) to account for variations in scanner sensitivity and coil profile. The quantitative SBF in mL/min/100g is derived from:

$$SBF=\frac{6000\;\cdot \;\lambda \;\cdot \;\left(SI_{control}-SI_{label}\right)\;\;\cdot \;\;e^{\frac{PLD}{T1_{blood}}}}{2\;\cdot \;\;\alpha \;\cdot \;T1_{blood}\cdot \;SI_{PD}\;\cdot \;\left(1-e^{\frac{-\tau }{T1_{blood}}}\right)}$$

Here λ is the blood-tissue partition coefficient (often set at 1.0 or 0.9 g/mL), a is the labelling efficiency (0.85), t is the label duration, and SIcontrol and SIlabel are the time-averaged signal intensities in the control and label images, respectively.^15^

ASL is uniquely suited to study the functional response of salivary glands to stimulation.^6^ Gustatory stimulation, such as with lemon juice, normally triggers a rapid increase in blood flow to support active secretion. In healthy glands, this response is swift and transient. However, ASL has shown that diseased states, such as Sjögren’s syndrome, display not only altered basal flow but also a fundamentally disordered kinetic profile after stimulation.^6^ This provides insights into the dysfunctional neurovascular coupling and microvascular resistance that contribute to xerostomia.

The differentiation of salivary gland tumors remains one of the most challenging tasks in head and neck radiology.^9^ The multiparametric integration of *diffusion-weighted imaging (DWI)* and *dynamic contrast-enhanced (DCE) MRI* offers a solution by assessing different aspects of tumor physiology.^16^

DWI measures the random movement of water molecules within the tumor microenvironment. The degree of restriction to this movement is quantified as the apparent diffusion coefficient (ADC). Tissues with high cellular density or specialized stroma restrict diffusion more than loosely organized tissues.^13^

The ADC is calculated using multiple b-values, which represent the strength and duration of the diffusion gradients^17^:

$$\text{S}(\text{b})=\text{S}(0){\text{e}}^{\text{-b}\;\text{ADC}}$$

By using a range of b-values (e.g., 0, 500, 1000 s/mm^2^), radiologists can effectively eliminate the “T_2_ shine-through” effect and obtain a pure measurement of tissue diffusivity. In salivary glands, ADC values clearly distinguish between mixoid-rich pleomorphic adenomas and cell-dense malignant or Warthin’s tumors.^13^

DCE-MRI involves the serial acquisition of T1-weighted images during the passage of a contrast bolus.^17^ This allows for the calculation of pharmacokinetic parameters using models such as the Tofts model^18^:

$$\text{dCt}(\text{t})/\text{dt}={\text{K}}^{\mathrm{trans}}\;{\text{C}}_{\text{p}}(\text{t})-{\text{k}}_{\text{ep}}\;{\text{C}}_{\text{t}}(\text{t})$$

Where C_t_(t) is the tissue contrast concentration, C_p_(t) is the arterial input function (plasma concentration), K_trans_ is the volume transfer constant (reflecting permeability and flow), and k_ep_ is the flux rate constant.

Analysing time-intensity curves (TIC) provides a semi-quantitative method to classify tumors based on their “wash-in” and “wash-out” patterns. For example, a “Type B” curve (rapid washin and rapid wash-out) is highly characteristic of Warthin’s tumors due to their high microvessel density and limited interstitial space.^10^

*MR Elastography* (MRE) is an innovative, non-invasive technique that objectively evaluates the biomechanical properties of tissues.^19^ While clinicians have long used physical palpation to detect hardness in the neck, MRE provides a “radiological palpation” with quantitative maps of tissue stiffness.^19^

MRE requires three main components: shear wave generation, displacement detection, and inversion into stiffness maps. Externally generated mechanical vibrations, typically at 25–60 Hz, are coupled into the parotid gland using a customized driver. A specialized phase-contrast MRI sequence then measures the displacement of tissue caused by these waves.^19^

The data are reconstructed to provide two primary surrogate parameters^20^:

1. Shear wave speed (SWS): correlates with tissue stiffness/elasticity and is measured in m/s or kPa
2. Loss Angle (ϕ): reflects tissue viscosity or fluidity, which is highly sensitive to inflammatory changes and interstitial fluid dynamics.

Studies have shown that parotid MRE is feasible and highly reproducible, providing normative stiffness values that serve as a baseline for detecting pathological changes such as fibrosis or malignant infiltration.^19^

## Quantitative results and clinical efficacy

The integration of advanced MRI technologies has yielded specific quantitative biomarkers that can reliably differentiate between healthy and pathological glandular states.^3^

*Hyposalivation* is defined as an objective decrease in saliva production, which may result from medications, radiotherapy, or autoimmune disease. Quantitative mapping has established specific cut-off values to identify these patients as presented in Table 1.^3^

**TABLE 1.** Diagnostic benchmarks for parotid gland dysfunction using multi-dynamic multi-echo (MDME) / synthetic MRI^3^

Parameter	Healthy controls (mean ± SD)	Hyposalivation group (mean ± SD)	Cut-off value	AUC
T1 relaxation (ms)	628.08	606.92	/	/
T2 relaxation (ms)	80.69 ± 6.42	91.85 ± 8.24	> 85.75	0.8131
Proton density (pu)	91.12	82.52	< 81.55	0.7588

1 AUC = area under the curve

**TABLE 2.** Resting and stimulated salivary blood flow (SBF) metrics *via* pseudo-continuous arterial spin labelling (pCASL)^6^

SBF parameter	Healthy glands	Sjögren’s syndrome glands
Base flow (mL/min/100g)	46.3 ± 9.0	59.2 ± 22.8
Stimulation response	47 ± 39%	74 ± 49%
SBF type	Type 1 (rapid)	Type 2/3 (delayed)

The significant prolongation of T_2_ times in the hyposalivation group serves as a physical indicator of parenchymal congestion and metabolic decline. This enables clinicians to differentiate between subjective xerostomia (the sensation of dry mouth) and objective glandular failure.^3^

ASL studies have revield unexpected findings in the perfusion of Sjögren’s syndrome (SS) glands.^6^ Despite the atrophy typically observed on conventional imaging, SS glands are mostly hyperemic in the resting state.^6^

This resting hyperaemia reflects active chronic inflammation and lymphocytic aggregation. The delayed and exaggerated response to gustatory stimulation indicates to impaired microvascular regulation and endothelial cell dysfunction, providing a functional biomarker that correlates with the severity of sicca symptoms.^6^

## Differential diagnosis of salivary tumors

Multiparametric MRI provides the data needed to distinguish specific tumor entities based on their biophysical signatures.^18^ The following Table 3 summarizes the diagnostic efficacy of combining conventional, diffusion, and perfusion MRI techniques for the preoperative characterization of salivary gland tumors (SGTs) based on recent clinical studies.

**TABLE 3.** Summarization of quantitative characterization of major salivary gland neoplasms^10,12,13,21^

Tumor type	ADC (×10^-3^mm^2^/s)	TIC curve pattern	K_ep_(min^-1^)	V_e_ (fractional vol)	Diagnostic features
Pleomorphic adenoma (PA)	High: 1.4–1.8	Type A (Progressive)	Low: ~ 0.69	High: ~ 0.65	High ADC + Type A curve is 95% specific for PA.
Warthin’s tumor (WT)	Low: < 0.9	Type B (Rapid washout)	High: ~ 6.2	Low: ~ 0.11	High K_ep_ and Type B curve differentiate WT from malignancy.
Lymphoma	Very low: 0.4–0.7	Type C (Plateau) or B	N/A (Typically low)	Variable	ADC < 0.7 is considered pathognomonic for lymphoma.
Malignant tumors (MT)	Moderate: 0.9–1.2	Type C (Plateau)	Moderate: ~ 1.72	Moderate: ~ 0.48	Type C curve is the most common finding for epithelial malignancies.

1 ADC = apparent diffusion coefficient; TIC curve = time-intensity curve

Using ADC alone often yields lower specificity for malignancies. However, multiparametric models (conventional MRI, ADC, and TIC) significantly increase diagnostic accuracy, with some studies reporting an area under the curve (AUC) as high as 0.96.^12^ This reduces the need for invasive procedures such as fine needle aspiration cytology (FNAC) in clearly benign cases and aids in surgical planning for malignancies.

Key conclusions:

- An ADC value >1.4 essentially excludes most malignancies and Warthin’s tumors. ADC values below 0.7 strongly indicate lymphoma over epithelial cancers or benign lesions.
- While ADC values for Warthin’s tumor and malignancy often overlap (the “gray area” of 0.9–1.2), the Kep and Ve values provide clear separation. A high Kep (> 2.44 min-1) is the most objective marker for Warthin’s tumor.
- Combining conventional MRI (shape and margins) with ADC and TIC curve analysis results in an accuracy rate of approximately 96%. Adding quantitative Tofts parameters (Ktrans, Kep, Ve) further increases the AUC to 0.96, particularly for difficult-to-distinguish parotid masses.

## Monitoring radiotherapy-induced injury

Head and neck radiotherapy (RT) is a common cause of permanent salivary gland damage.^1^ Advanced imaging is crucial for early detection and prevention of chronic xerostomia.^1,22^

Research on patients with nasopharyngeal carcinoma has shown that significant changes occur in the parotid glands as early as five weeks into the radiotherapy course.^16^ There is a measurable increase in T_2_ relaxation times (reaching 121 ± 20 ms compared to 96 ± 12 ms in controls)^7^ accompanied by an increase in the fat fraction (FF).

The elevation in T_2_ values serves as an objective biomarker for acute tissue inflammation and oedema. The simultaneous rise in FF indicates the onset of parenchymal replacement by fatty tissue, even before volume loss is evident on conventional scans. Technical findings indicate that T2 values continue to rise FF indicates the onset of parenchymal replacement by fatty tissue, even before volume loss is evident on conventional scans. Technical findings indicate that T2 values continue to rise even after the conclusion of radiotherapy, suggesting that parenchymal damage is a dynamic, progressive process rather than a static injury.^15^

IVIM MR, ADC and other modern imaging techniques can be used to assess or predict a patient’s risk of developing severe xerostomia after RT.^1,5,12,22–25^ Statistical features such as the parameter informational measure of correlation 1 (IMC 1) have achieved a diagnostic accuracy of 75% in predicting post-RT dryness from ADC maps.^26^ This allows for the optimization of parotid-sparing radiotherapy plans (e.g. intensity-modulated radiation therapy, IMRT), where clinicians can identify specific “functional hotspots” within the gland to be prioritized for dose protection.^8^

## Example study of advanced imaging methods used in therapeutic response to Hyperbaric Oxygen Therapy

In the study of Monitoring the effect of HBO on post-radiation xerostomia, conducted at MF Ljubljana, advanced multiparametric MR analysis (T_2_ mapping and ASL) was used to monitor the therapeutic effect of HBO.^7^

HBOT is an established treatment for late radiation-induced tissue toxicity, targeting the underlying vascular and cellular deficits.^27^ Advanced MRI is the method to non-invasively monitor the tissue’s restorative response.

The clinical improvement observed after HBOT is supported by complex biological transitions that can be tracked radiologically:

- Revascularization: elevated oxygen levels in the hyperbaric chamber (often reaching PaO2 > 1000 mmHg) stimulate angiogenesis through upregulation of vascular endothelial growth factor (VEGF) and recruitment of stem cells.^28^ This addresses the “hypovascular” component of radiation injury.
- TGF-beta regulation: HBOT has been experimentally shown to attenuate the expression of genes involved in the TGF-ß pathway, which is critical for inhibiting the progression of chronic fibrosis in irradiated glands.^29^
- Chronic oedema resolution: by promoting revascularization and reducing inflammation, HBOT leads to stabilization or reduction of the elevated T_2_ values found in irradiated glands.^7^
- In our study, T_2_ mapping was used to monitor the parotid glands of patients following 20 sessions of daily HBOT at 2.5 ATA.^7^

The reduction in mean T_2_ values after HBOT (as seen in Table 4) indicates normalization of tissue hydration and a decrease in chronic inflammation. Importantly, these MRI changes were strongly correlated with clinical improvements, including increased unstimulated whole salivary flow and improved salivary pH and buffering capacity. This confirms that quantitative MRI can serve as a robust, objective indicator of therapeutic success in regenerative medicine.^7^

**TABLE 4.** Evolution of parotid T2 values during restorative hyperbaric oxygen therapy (HBOT)^7^

Gland group	Mean T2 before HBOT (ms)	Mean T2 after HBOT (ms)	Control group T2 (ms)
Ipsilateral (high dose)	121 ± 20	113 ± 16	96 ± 12
Contralateral (low dose)	107 ± 21	103 ± 14	96 ± 12

**TABLE 5.** Summary of information provided by different salivary imaging modalities

Modality	Spatial resolution	Functional parameters	Advantages	Limitations
Scintigraphy	Low	Global excretion rate	Well-established; measures total function	Ionizing radiation; poor anatomical detail
MR sialography	High	Ductal anatomy	Non-physiological; excellent for stricture detection	Does not measure perfusion
T2 mapping	High	Water content; oedema	Quantitative; non-contrast	Requires advanced software
ASL MRI	Medium/high	Salivary blood flow (SBF)	Completely non-invasive; measures microcirculation	Sensitive to patient motion
DWI / ADC	Low/medium	Cellularity; microstructure	Quantitative; differentiates between tumors	Overlapping values; low resolution
DCE-MRI	High	Vascularity; permeability	Excellent characterization of tumor kinetics	Requires gadolinium; invasive (IV)
MR elastography (MRE)	High	Tissue stiffness; fibrosis	Non-invasive assessment of biomechanical properties	Requires additional hardware

1 ADC = apparent diffusion coefficient; ASL = arterial spin labelling; DCE = dynamic contrast-enhanced; DWI = diffusion-weighted imaging

**FIGURE 1.**
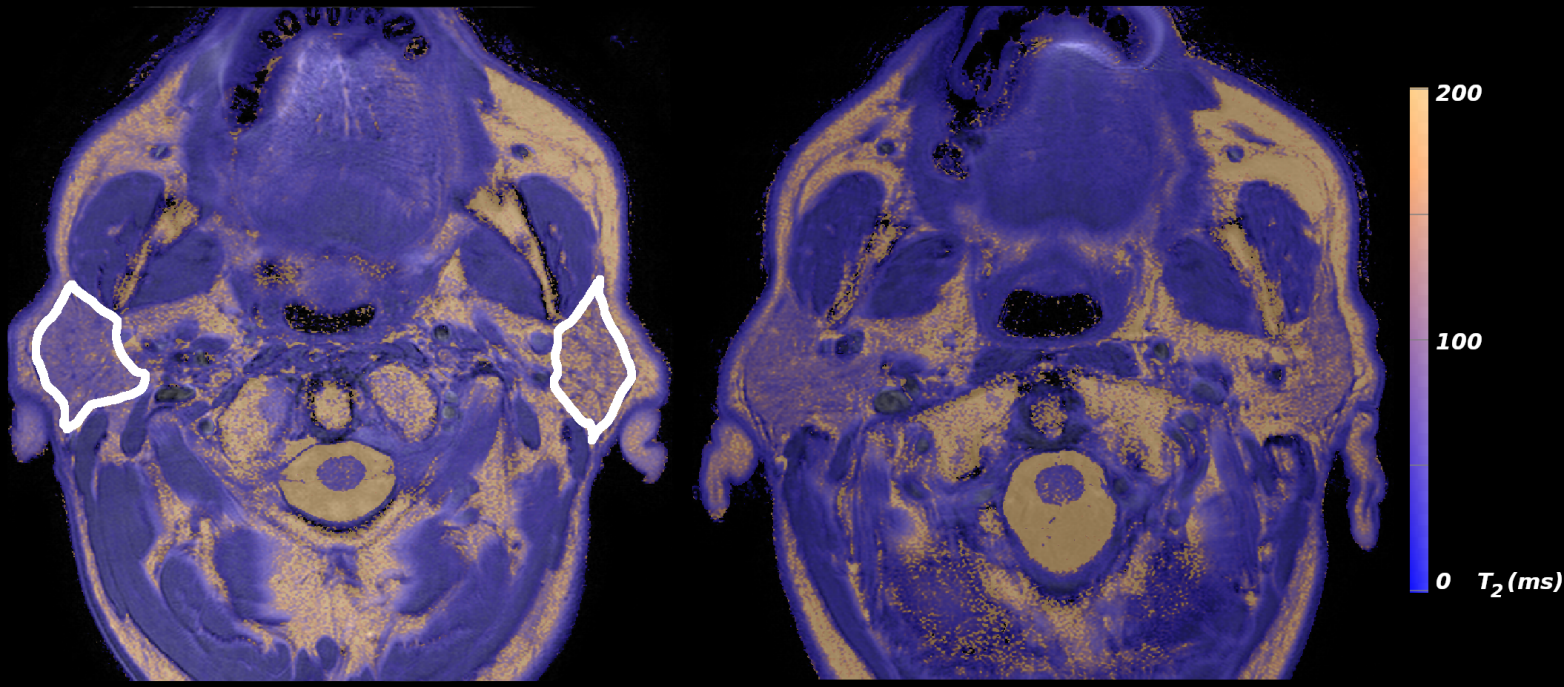
Representative T_2_ relaxation time maps of the parotid glands in a patient with a head and neck malignancy, acquired at baseline (left) and following hyperbaric oxygen (HBO) therapy (right).

As seen in Figure 1, following HBO therapy, there is a notable increase in signal homogeneity and a reduction in inter-glandular variance between the left and right parotid glands compared to the pre-therapeutic scan.

ASL analysis showed that in irradiated glands, changes occurred after HBOT, with the most prominent differences observed at intermediate to long PLDs, particularly after gustatory stimulation.

**FIGURE 2.**
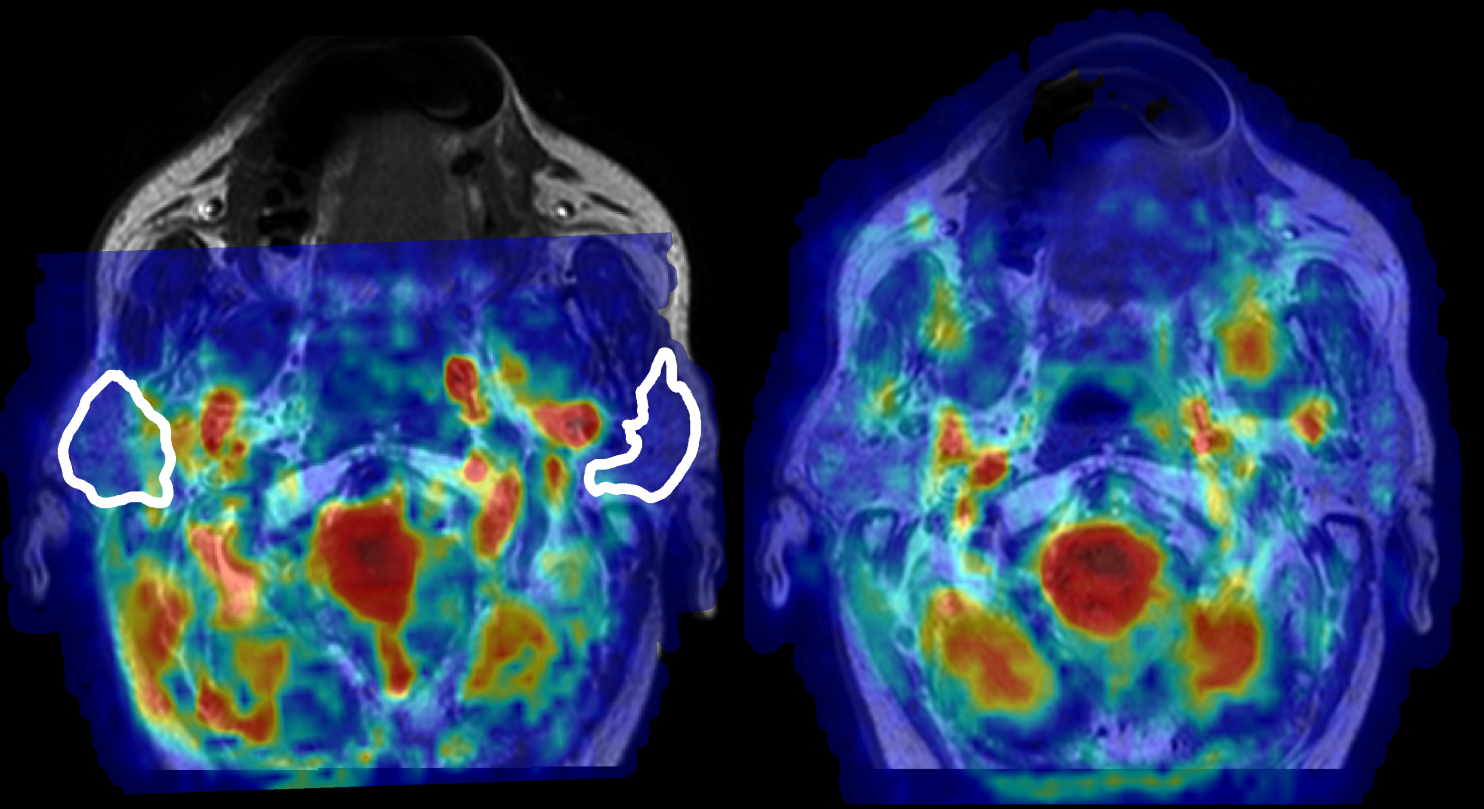
Qualitative assessment of arterial spin labelling (ASL) perfusion maps pre- and post-hyperbaric oxygen (HBO) therapy following lemon juice stimulation.

Note the bilateral perfusion dynamics within the parotid glands in Figure 2. Post-HBO intervention, the right parotid gland demonstrates a significant increase in perfusion signal intensity. This indicates a restoration of microvascular flow, achieving greater symmetry with the healthy contralateral (left) parotid gland.

## Comparison with traditional modalities

The role of advanced MRI should be considered complementary to established clinical and nuclear medicine tools, as each provides different information on glandular health.

Scintigraphy with ^99m^Tc-pertechnetate has long been the gold standard for global functional assessment.^30^ It measures the ability of all glands to concentrate and secrete the isotope simultaneously. However, it is limited by low spatial resolution, poor anatomical detail, and exposure to ionizing radiation.^30^ Advanced MRI mapping offers higher sensitivity for early localized changes and provides anatomical context that scintigraphy lacks.

MR sialography, particularly with heavily T2-weighted 3D sequences (T2-3D-DRIVE), provides unmatched spatial resolution of the ductal system.^31^ It uses saliva as a natural contrast agent to visualize strictures and dilation up to second-order branches.

Integrating these techniques enables a comprehensive “imaging labial biopsy” approach, combining structural, ductal, and microvascular data to refine clinical decision-making.

## Future directions and precision radiology

The field of salivary gland imaging is moving toward an automated, AI-driven framework that integrates imaging with other diagnostic data.^32^

The quantitative nature of T_2_, ADC, and pCASL maps makes them ideal candidates for analysis by artificial intelligence.^33^ Machine learning algorithms can identify subtle textural patterns that reflect early disease stages or predict tumor aggressiveness.^24,26^ This approach can integrate pixellevel data into a single prognostic model, assisting radiologists predict treatment outcomes with a level of precision that was previously unattainable.

Future research is expected to focus on combining multiparametric MRI findings with molecular biomarkers found in saliva. For example, integrating MRE-derived stiffness data with levels of salivary VEGF or inflammatory cytokines could provide a comprehensive view of the glandular microenvironment. This integration is essential developing targeted therapies and personalized monitoring of chronic salivary conditions.

A significant hurdle for the widespread adoption of advanced MRI is the lack of standardized protocols across different hardware vendors.^19^ Future efforts must focus on establishing international normative benchmarks for parotid T_2_ and SBF values to ensure that findings from one center can be reliably compared to another. The development of specialized head and neck coils and the reduction of motion artifacts will also be crucial for bringing these technologies into daily dental and surgical practice.^34^

## Discussion and nuanced perspective

The transition from qualitative morphological imaging to quantitative physical mapping represents the most significant change in salivary gland diagnostics in decades.^35^ One of the key insights provided by advanced mapping is the resolution of the discrepancy between subjective and objective symptoms in patients with xerostomia.^22^ T_2_ mapping offers the first physical evidence of parenchymal congestion, reflected in elevated ms values, that correlates with the patient’s sensation of dry mouth. This highlights the role of T_2_ as a marker not only of water content, but also of cellular health and micro-environmental integrity.^16^

The resting hyperemia identified by ASL in Sjögren’s syndrome establishes a causal link between the histopathological finding of lymphocytic infiltration and the clinical symptom of gland dysfunction.^6^ This hyperemia is likely a compensatory response to the inflammatory demand, but it results in microvascular regulatory failure.^6^ This suggests that ASL could serve as an earlystage screening tool to detect SS before irreversible structural damage occurs, enabling more timely pharmacological intervention.^8^

In tumor characterization, the high ADC values and Type A kinetic curves of pleomorphic adenomas provide a definitive signature that can prevent unnecessary surgical escalation or biopsy-related complications.^21^ In contrast, the intermediate ADC values and Type C patterns of malignant tumors serve as clear warning signs that can prompt immediate referral for radical management.^21^ The addition of MRE-derived stiffness offers an extra layer of security, as malignant lesions consistently exhibit a higher shear elastic modulus than benign counterparts.^19^

The restorative role of HBOT, long debated in clinical oncology, is now firmly supported by objective parametric data.^27^ The demonstration of a significant reduction in T_2_ relaxation times along with improved salivary flow provides conclusive physical evidence of tissue regeneration and reduced chronic inflammation.^7^ This confirms that HBOT is not merely a palliative measure for symptoms but a biological intervention that modifies the glandular microenvironment toward restoration.^7^

Despite these clear advantages, technical implementation remains sensitive to hardware and patient factors.^15^ pCASL efficiency depends heavily on arterial flow velocity and precise shim settings in the labelling plane.^15^ Similarly, T_2_ values can be influenced by the choice of fitting model (mono-*vs*. multi-exponential) and the number of echoes acquired. These challenges must be addressed through technical refinement and the development of robust, user-independent processing software.

## Conclusions

Advanced MRI technologies, particularly quantitative T_2_ mapping and pCASL perfusion, represent an essential evolution in the structural and functional assessment of salivary glands. By providing objective, reproducible physical benchmarks, these methods overcome the inherent subjectivity of conventional grayscale imaging. Integrating multiple parameters, as cellularity (ADC), perfusion (SBF), hydration (T_2_), and stiffness (MRE), into a comprehensive diagnostic profile enables early detection of pathology, precise differentiation of complex tumors, and the robust monitoring of restorative responses following radiotherapy and hyperbaric oxygen therapy. As these technologies transition from experimental research to routine clinical standardized workflows, they will form the technological foundation of precision radiology in head and neck medicine, ultimately improving therapeutic precision and quality of life for patients worldwide.
